# Histopathology of *Aculeastrum americanum* on *Rubus idaeus* and insights into the chloroplast-pathogen interaction

**DOI:** 10.3389/fpls.2025.1630100

**Published:** 2025-10-14

**Authors:** Lucas Henrique Santos Barbosa, Ulla Neumann, Ton Timmers, Tonni Grube Andersen, Beatriz Appezzato-da-Glória

**Affiliations:** ^1^ Plant Anatomy Laboratory, Department of Biological Sciences, “Luiz de Queiroz” College of Agriculture, University of São Paulo, Piracicaba, Brazil; ^2^ Central Microscopy, Max Planck Institute for Plant Breeding Research, Cologne, Germany; ^3^ Department of Plant-Microbe Interactions, Max Planck Institute for Plant Breeding Research, Cologne, Germany

**Keywords:** callose, chloroplast, haustoria, late leaf rust, *Pucciniastrum americanum*, raspberry, starch, *Thekopsora americana*

## Abstract

**Introduction:**

Raspberry late leaf rust, caused by *Aculeastrum americanum* (Farl.) M. Scholler & U. Braun has been reported in several countries. All aerial parts of the plant can be infected, with the primary symptoms of this disease being powdery yellow spots. Lesions reduce leaf gas exchange and lead to early defoliation. Moreover, infected fruits become unmarketable, resulting in severe yield losses. Despite the growing threat of this rust, the histopathology of *A. americanum* on raspberry remains poorly understood, particularly on *Rubus idaeus* L., one of the widely cultivated and economically important raspberry species.

**Methods:**

This study provides a detailed analysis of the infection, colonization, and reproduction processes of *A. americanum* on raspberry leaves, using light microscopy (bright field and fluorescence), confocal laser scanning microscopy, as well as scanning and transmission electron microscopy.

**Results and discussion:**

Our findings provide the first microscopic evidence, in rust fungi, of the formation of two haustoria within a single host cell. Chloroplasts were observed in close association with the *A. americanum* haustorium, and underwent a series of alterations, that help to explain the drastic reduction in leaf gas exchange during late leaf rust infection. Although infected leaves produce defense substances, such as callose and phenolic compounds, raspberry leaves are unable to prevent successful colonization. The occurrence of cell collapses and necrosis, together with the ultrastructural alterations, likely contributes to the early defoliation observed in raspberry plants infected by *A. americanum*. This study provides novel insights into chloroplast-pathogen interactions, highlighting previously unrecognized aspects of chloroplast alterations during late leaf rust infection. Nevertheless, further investigations are required to deepen our understanding of this relationship in rust fungi as well as in other biotrophic pathogens.

## 1 Introduction

Raspberry late leaf rust, caused by *Aculeastrum americanum* (Farl.) M. Scholler & U. Braun (syn. *Pucciniastrum americanum* (Farlow) Arthur and syn. *Thekopsora americana* (Farl.) Aime McTaggart), originated in North America (Delisle-Houde et al., 2020; Rebollar-Alviter et al., 2003; Scholler et al., 2022). The disease, which is now widely distributed, has been reported in Canada (Luffman and Buszard, 1989), Mexico (Rebollar-Alviter et al., 2003), Argentina (Lucero et al., 2008) and Brazil, where it is the most important disease affecting raspberry production (Figueiredo et al., 2003). More recently, it was reported in New Zealand (Hofer et al., 2025), where *A. americanum* has been classified as a quarantine organism of biosecurity concern, due to red raspberries representing a key commercial crop (Bleach, 2023).

Leaves, stems, and fruits at all developmental stages may be infected by *A. americanum*, with the primary symptoms of this disease being powdery yellow spots (Nelson, 2011), which correspond to reproductive structures called uredinia (Dias et al., 2023). Lesions on leaves cause reduced leaf gas exchange (Ribeiro and Spósito, 2022) and lead to early defoliation (Nelson, 2011; Hofer et al., 2025). In highly susceptible cultivars, plants are often reduced to leafless stems. Infected fruits become unmarketable due to the appearance of yellow spots, premature ripening and subsequent rotting (Nelson, 2011), causing severe yield losses (Lucero et al., 2008). Therefore, more efforts are needed to help address and mitigate the impact of this disease. Notably, the histopathology of *A. americanum* on raspberry remains poorly understood, particularly on *Rubus idaeus* L., one of the widely cultivated (Davik et al., 2022) and economically important raspberry species (Foster et al., 2019).

Histopathological studies of plant-pathogen interactions are essential for tackling emerging rust diseases, particularly those affecting food crops, as they provide insights into structural changes in host tissue and form the basis for understanding pathogen development and disease epidemiology (Dias et al., 2023; Esmail et al., 2019; Gonçalves et al., 2023; Morales et al., 2024; Rasera et al., 2024).

Rust fungi are obligate biotrophic pathogens (Duplessis et al., 2021) that form specialized infection structures to interact with their hosts. These include appressoria, which are crucial for host penetration (Allen, 1991), and haustoria, which facilitate intimate host-pathogen interactions and are responsible for nutrient acquisition from host cells (Mendgen et al., 2000; Mendgen and Hahn, 2002; Voegele and Mendgen, 2003).

During rust infection, chloroplasts, which are a key component of early immune responses (De Torres Zabala et al., 2015), may undergo alterations such as structural disorganization, including the de-stacking of thylakoids, as reported in *Triticum aestivum* leaves infected by *Puccinia striiformis* (Aldesuquy et al., 2000), and chloroplast degeneration (Nogueira Júnior et al., 2017). In grapevines infected by *Phakopsora euvitis*, chloroplasts in infected cells are transformed into gerontoplasts, which may explain early defoliation (Rasera et al., 2019). Furthermore, starch dynamics in leaves are reported to be altered during rust infections (Chou et al., 2000; Nogueira Júnior et al., 2017; Scholes and Farrar, 1987), leading to starch accumulation at infection sites at the expense of other plant regions (Long et al., 1975; Cheaib and Killiny, 2025), as observed in grapevine rust, where leaf starch accumulation occurs at the expense of root reserves (Nogueira Júnior et al., 2017). To date, the only histological study addressing the infection process of *A. americanum* in raspberry leaves was conducted by Dias et al. (2023), who reported stomatal penetration and the formation of a single haustorium per host cell. However, broader structural and ultrastructural analyses of host responses are still lacking.

In this study, we aim to fill the existing gap in the histopathological understanding of the interaction between *A. americanum* and *Rubus idaeus*, which remains limited, particularly given the devastating effects of the disease on raspberry plants. By characterizing the processes of infection, colonization, and reproduction of *A. americanum* on raspberry leaves, our goal is to provide a more detailed view of the pathogenesis of this fungus. Additionally, we investigate the interactions between chloroplasts and the pathogen, further expanding knowledge on how the disease affects the plant’s physiology. This research may support future molecular studies focused on the mechanisms of host resistance.

## 2 Materials and methods

### 2.1 Biological material

Raspberry seedlings (*Rubus idaeus* cv. Heritage) were grown in pots (7 L) containing sterilized substrate (clay soil and sand, 1:2) with a granulometry of 10% clay and 70% sand. Plants were cultivated under greenhouse conditions (25 ± 5 °C; relative humidity 60 to 90%) and irrigated daily with approximately 300 mL of water per pot. Weekly, each pot received 50 mL of a liquid fertilizer solution containing NPK (8:3:8) and micronutrients (Forth Jardim^®^). A voucher specimen was deposited in the ESA Herbarium under accession number 157660.

The experiment was conducted using *Aculeastrum americanum* monopustular isolate (GenBank MW039448) obtained from *R. idaeus* (Ribeiro and Spósito, 2022). To maintain the inoculum, the fungus was multiplied in *R. idaeus* cv. Heritage, kept in a greenhouse. For inoculation with the fungus *A. americanum*, the collected urediniospores were suspended by adding distilled water. In treatments involving inoculation, all fully expanded leaves per plant were inoculated by spraying a suspension of 5x10^4^ urediniospores mL^-1^ with 0.05% Tween 20 on both leaf surfaces until dew point, with a spray nozzle (NS 19/26; Lenz) coupled to a portable 116 electric atomizing sprayer at 1 bar (MA 2057; Marconi). The leaves of non-inoculated plants were sprayed with distilled water. All plants (inoculated and non-inoculated) were kept for 24 hours in the dark, in a humid chamber at 23 °C (Ribeiro and Spósito, 2022), and then taken to the greenhouse. Leaf samples were collected from the middle third of the plants at 1 to 28 days after inoculation (DAI) of 10 plants, with five plants inoculated with *A. americanum* and five plants that were not inoculated (control).

### 2.2 Bright field microscopy

For the BM analyses, leaf samples of 1 cm^2^ were fixed in Karnovsky solution (Karnovsky, 1965) for 48 h. During this period, the samples were five times taken to a vacuum pump to remove the air from the tissues and then dehydrated in a graded ethanol series (10–100%). After dehydration, the samples were embedded in hydroxy-ethyl-methacrylate (Leica Historesin, Heraeus Kulzer, Hanau, Germany). The blocks were sectioned in a rotary microtome (Leica RM2245, Leica Biosystems, Heidelberg, Germany) at 5 μm thickness, and the sections were stained with toluidine blue (Sakai, 1973). To detect starch grains, some leaf sections were treated with zinc chloride iodine (Strasburger, 1913) or Lugol (Gerlach, 1984). Samples were also fixed in ferrous sulfate solution in formalin for detection of phenolic compounds (Johansen, 1940). The sections were analyzed under a Leica DMLB microscope (Leica Microsystems) and images were captured using a Leica DFC310 FX camera.

### 2.3 Fluorescence microscopy

For FM analysis, sample processing including sectioning followed the protocol outlined in the preceding section. Some tissue sections were stained with WGA-Alexa Fluor 488 in phosphate-buffered saline (PBS) pH 7.2 for 20 minutes and mounted in distilled water. Fluorescence microscopy (DM 5500; Leica) was used to observe the WGA-Alexa Fluor 488 signal, either with a 5 L filter (460–500 nm excitation; 515–585 nm emission) alone (Marques et al., 2018) or in combination with a DAPI filter (340–360 nm excitation; LP 425 nm emission). For chloroplast autofluorescence analysis, sections were mounted on slides in distilled water and examined using a Leica DMLB microscope equipped with a fluorescence light source (ebq 100; Leica) and a digital camera (DFC310 FX; Leica). Fluorescence images were acquired using D filter set with 365 nm excitation and 420 nm emission wavelengths.

### 2.4 Confocal laser scanning microscopy

The leaf samples (0.8 cm^2^) for CLSM were fixed in 0.15% (w/v) trichloroacetic acid in ethanol/chloroform 4:1 (v/v). The fixation/destaining solution was renewed regularly over a period of one week until the samples were completely cleared. Then, the leaf tissue fragments were washed three times in fresh PBS pH 7.4. Samples were treated in 1 M KOH solution at 37 °C for 1 h, followed by washing in PBS pH 7.4 containing 0.1% Triton X-100. Afterwards, the samples were vacuum-infiltrated in a staining solution (20 μg mL^−1^ WGA-Alexa Fluor 488, 50 μg mL^−1^ propidium iodide, 20 μg mL^−1^ bovine serum albumen [BSA] and 0.1% Triton X-100 in PBS pH 7.4) and incubated overnight at 4 °C. The stained samples were washed in fresh PBS pH 7.4 containing 0.1% Triton X-100 and finally placed in PBS pH 7.4 containing 25% glycerol (Morales et al., 2023). Confocal laser scanning microscopy was performed using a Zeiss LSM 980 system. Image acquisition settings for the two fluorophores were as follows: excitation 488 nm, emission 499–542 nm for WGA-Alexa Fluor 488, excitation 561 nm, emission 605–649 nm for propidium iodide.

High-resolution images were obtained in sequential scan mode with a Leica SP8 FALCON-DIVE in multiphoton mode using 900 nm light. The Alexa Fluor was excited at 488 nm and detected at 493–547 nm and the propidium iodide was excited at 552 nm and detected at 580–650 nm using the PMT detector. The Leica application software LAS X 3d module was used for depth color-coding and reconstruction of z-stacks.

### 2.5 Scanning electron microscopy

For the SEM analyses, samples of approximately 1 cm^2^ of inoculated leaves were fixed in Karnovsky solution (Karnovsky, 1965) for 48 h. The samples were subsequently dehydrated in an ethanol series from 10% to 100%, critical point dried with CO_2_ (Horridge and Tamm, 1969), mounted on aluminium stubs and coated with a gold layer (30–40 nm) using a Balzers SCD 050 sputter coater. Observations and photomicrographs were obtained using a Zeiss LEO 435 VP SEM, which was operated at 20 kV, and scale bars were directly printed on the electron micrographs generated.

### 2.6 Transmission electron microscopy

The samples of control and inoculated leaves for TEM analyses were fixed in 2.5% glutaraldehyde and 2% paraformaldehyde in 0.1 M sodium cacodylate buffer, pH 7.2, supplemented with 0.025% CaCl_2_ (wt/vol) and maintained in a vacuum pump for air removal. Samples were rinsed three times for 10 min in 0.1 M sodium cacodylate buffer (pH 6.9), then post-fixed for 1 h at room temperature with 0.5% OsO_4_ in 0.1 M sodium cacodylate buffer, pH 7.2, supplemented with 0.15% potassium ferricyanide. Subsequently, samples were rinsed thoroughly with MilliQ ELIX water and dehydrated in an ethanol series from 10% to 100%, gradually transferred to acetone, and then gradually embedded over two days into Araldite 502/Embed 812 resin using the EMS Lynx II embedding machine. Resin polymerization was performed at 60 °C for 48 h. The blocks were sectioned using a Reichert-Jung ultramicrotome. Ultrathin sections (70–90 nm) were cut using a diamond knife and deposited on nickel slot grids coated with 0.5% formvar film. For post-sectioning contrast, sections were incubated at room temperature for one minute in uranyl acetate replacement (UAR-EMS, Science Services, Germany, catalogue number E22405), followed by 3% lead citrate (Science Services Germany, catalogue number DM22410) for one minute.

For immunogold labelling of callose, sections were blocked for 30 min in a 1:30 dilution of goat normal serum in TRIS buffer (20 mM TRIS, 225 mM NaCl, 20 mM NaN3, pH 6.9) supplemented with 1% (wt/vol) BSA (TRIS-BSA). After three washes for 10 min in TRIS-BSA, sections were incubated in a 1:100 dilution of the primary antibody (anti-ß-1,3-glucan; Biosupplies Australia, catalogue number 400-2) at 4 °C overnight. The sections were washed four times for 10 minutes in TRIS-BSA and, subsequently, sections were incubated with a 1:20 dilution of the corresponding secondary antibody (goat anti-mouse) conjugated to 10 nm colloidal gold particles (bbi EM.GAM10) at room temperature for 1 h. After thorough washing with first TRIS-BSA and then filter-sterilized, demineralized water, micrographs were taken with a Hitachi HT7800 TEM operating at 100 kV and equipped with an EMSIS XAROSA camera.

## 3 Results

### 3.1 Infection, colonization, and chloroplasts at the host-pathogen interface

**Figure 1:**
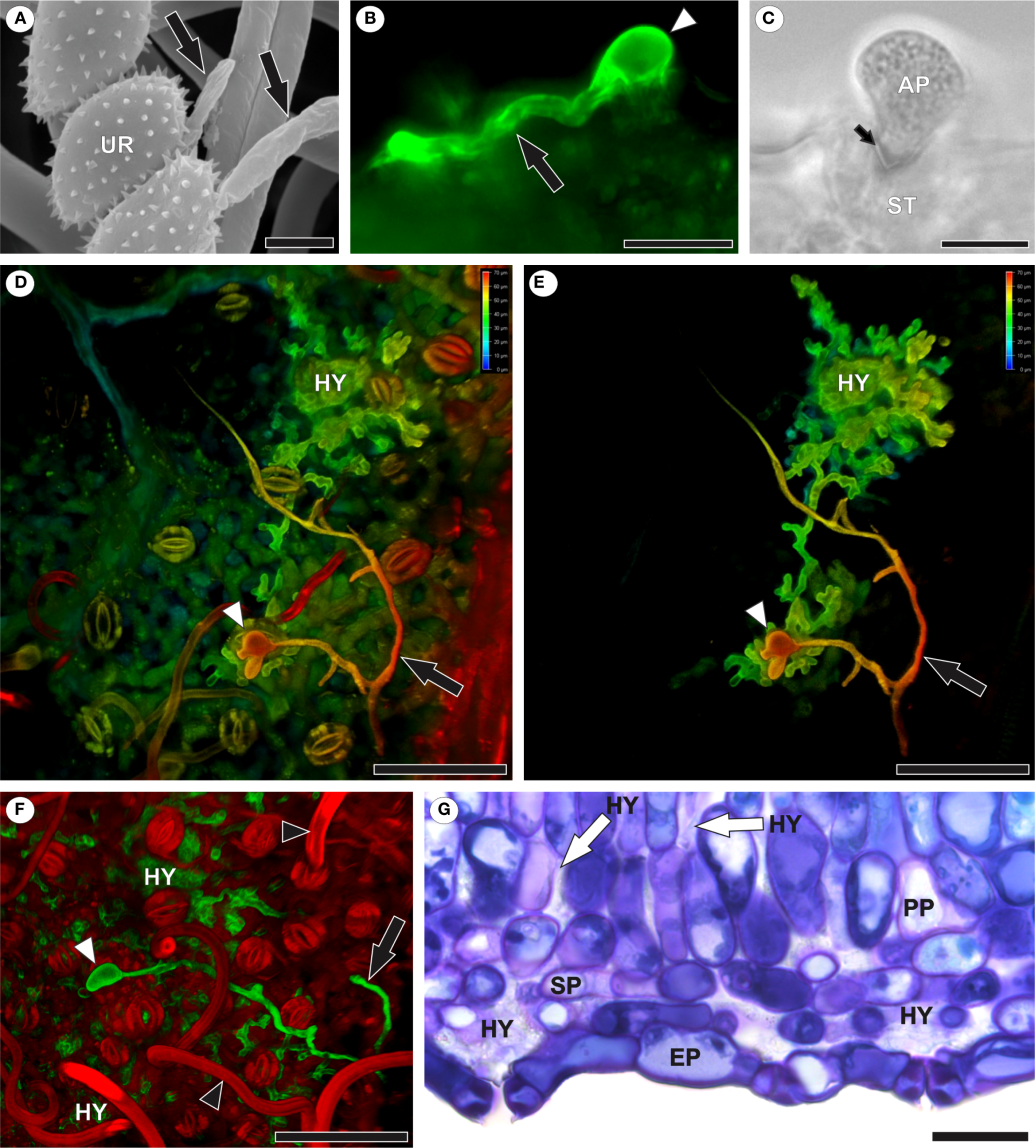
Infection and colonization of *Aculeastrum americanum* of raspberry leaves. **(A)** Scanning electron micrograph showing urediniospore germination with germ tubes (black arrows) at 1 day after inoculation (DAI). **(B)** Fluorescence micrograph showing germ tube (black arrow) and appressorium (white arrowhead) over stomata stained with WGA Alexa Fluor 488 at 10 DAI. **(C)** Bright field image of fungal penetration through the ostiole at 10 DAI. **(D–F)** Confocal images showing branched germ tubes (black arrows), appressoria (white arrowheads), and hyphae in mesophyll intercellular spaces at 7 DAI. False colors in 3D z-stack reconstructions **(D, E)** represent depth information (red to blue, focus from top to bottom of stack). **(F)** Highlights extensive colonization. Note trichomes (black arrowhead). False colors in channel overlay image **(F)** shows WGA Alexa Fluor 488 (green) and propidium iodide (red). **(G)** Bright field micrograph of a leaf cross-section showing hyphal colonization, especially in substomatal chambers. AP, appressorium; EP, epidermis; HY, hyphae; PP, palisade parenchyma; SP, spongy parenchyma; UR, urediniospore. Scale bars: 5 μm **(A)**; 10 μm **(C)**; 20 μm **(B, G)**; 50 μm **(D–F)**.

After germination of *A. americanum* urediniospores (**Figure 1A**), one or more elongated germ tubes, which may be either branched or unbranched (**Figures 1B, D**), formed on the abaxial leaf surface. Appressorium formation occurred exclusively over the stomata (**Figures 1C–E**), through which the pathogen penetrated. Hyphae developed in the intercellular spaces of the mesophyll, particularly in the substomatal chambers (**Figures 1D–G**).

**Figure 2:**
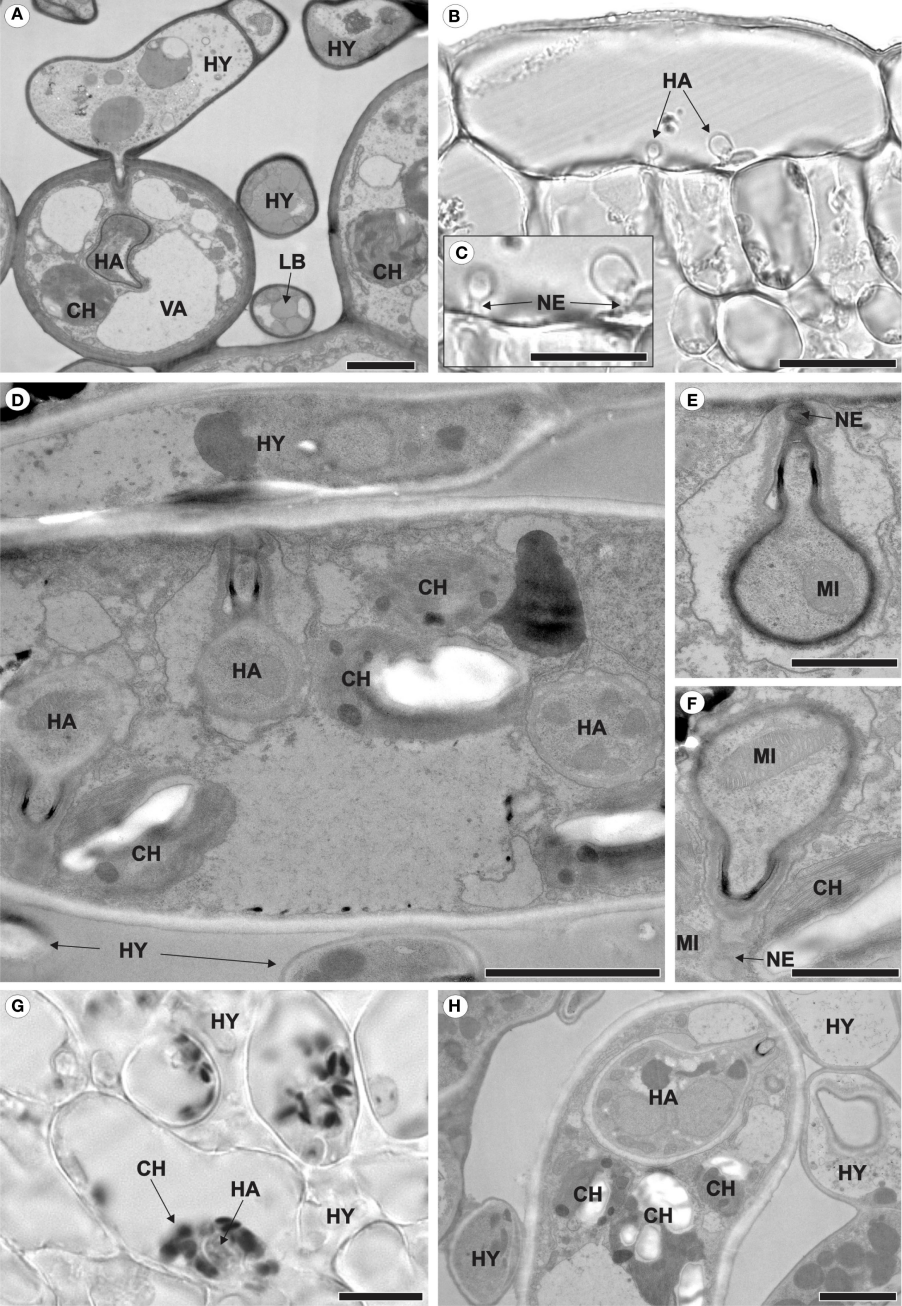
Intracellular growth of *Aculeastrum americanum* on/in raspberry leaves at 7 days after inoculation (DAI). **(A, D–F, H)** Transmission electron micrographs. **(B, C, G)** Bright field micrographs. **(A)** Fungal hyphae colonizing the mesophyll and producing haustoria inside spongy parenchyma cells. **(B)** Two haustoria inside an epidermal cell. **(C)** Detail of the haustoria shown in **(B)**. **(D)** Two haustoria of the fungus inside the same palisade parenchyma cell. **(E, F)** Details of the haustoria shown in **(D)**, with **(E)** showing the middle haustorium and **(F)** the left haustorium. **(G, H)** Chloroplasts surrounding the haustorium. In **(G)**, the dark/black areas represent starch within the chloroplasts after reaction with Lugol’s solution at 28 DAI. CH, chloroplast; HA, haustoria; HY, hyphae; LB, lipid bodies; MI, mitochondria; NE, haustorial neck; VA, vacuole. Scale bars: 1 μm **(E, F)**; 2 μm **(A, D, H)**; 20 μm **(B, C, G)**.

**Figure 3:**
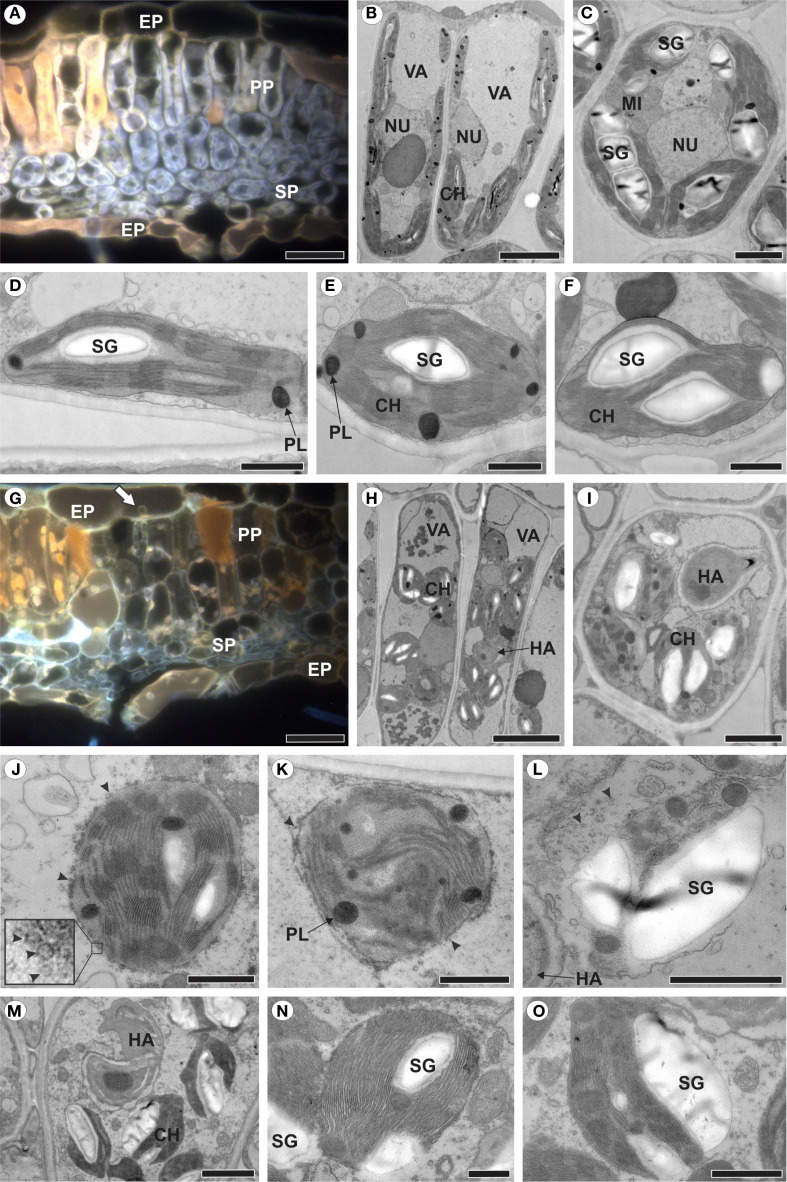
Healthy raspberry leaves **(A–F)** and those colonized by *Aculeastrum americanum*, exhibiting plastid alterations **(G–O)**. **(A, G)** Fluorescence micrographs acquired using a D filter (excitation at 355–425 nm, long-pass emission at 470 nm). **(B–F, H–O)** Transmission electron micrographs. **(A)** Cross section of healthy leaf showing autofluorescence of chloroplasts (light grey) and phenolic compounds (orange). **(B)** Palisade parenchyma cells and **(C)** spongy parenchyma cells of non-inoculated plants. **(D)** Chloroplast of palisade parenchyma cell. **(E, F)** Chloroplasts of spongy parenchyma cells. **(G)** Cross section of infected leaf showing no chloroplast autofluorescence at 14 days after inoculation (DAI). Note presence of haustoria in epidermal cell (arrow). **(H)** Palisade parenchyma cells and **(I)** spongy parenchyma cell of inoculated plants. **(J–L)** Plastid changes at 7 DAI with chloroplast membrane showing vesiculation (**J**, arrowheads), followed by de-stacking of thylakoids **(K)**, resulting in plastid degeneration and release of starch grain into the cytosol **(L)**. **(M-O)** Release of starch grains with plastid envelope partially disrupted. CH, chloroplast; EP, epidermis; HA, haustorium; MI, mitochondria; NU, nucleus; PL, plastoglobuli; PP, palisade parenchyma; SG, starch grain; SP, spongy parenchyma; VA, vacuole. Scale bars: 500 nm **(N, O)**; 1 μm **(D–F, J–L)**; 2 μm **(C, I, M)**; 5 μm **(B, H)**; 20 μm **(A, G)**.

The pathogen grew intracellularly, forming a single haustorium (**Figure 2A**) or, notably, two haustoria per cell (**Figures 2B–D**). Each haustorium comprised a neck and a haustorial body (**Figures 2C–E**). The occurrence of two haustoria per cell was observed in both epidermal (**Figure 2B**) and palisade parenchyma (**Figure 2D**) cells. In infected cells, where haustoria were present, chloroplasts were positioned around the haustorium (**Figures 2G, H**), whereas in uninfected cells, such as those in non-inoculated leaves or inoculated leaf cells lacking haustoria, chloroplasts were aligned parallel to the mesophyll cell walls (**Figures 3A–C**).

### 3.2 Plastids degenerate in colonized areas

To identify plastid changes in raspberry leaves colonized by *A. americanum*, non-inoculated leaves were first analyzed (**Figures 3A–F**). In mesophyll cells, the chloroplasts showed autofluorescence (**Figure 3A**) and a discoid shape, being generally more elongated in the palisade parenchyma cells (**Figures 3B, D**) compared to the spongy parenchyma chloroplasts (**Figures 3C, E, F**), with well-organized and stacked thylakoid membranes, containing starch grains and plastoglobules (**Figures 3B–F**).

In contrast, in leaves colonized by *A. americanum*, the chloroplasts did not exhibit autofluorescence (**Figure 3G**). When haustoria were present inside the cell, the chloroplasts underwent a series of alterations (**Figures 3H–O**). The chloroplast outer membrane displayed vesiculation (**Figures 3J, K**), which progressed to the complete structural disintegration of the plastid (**Figure 3L**). Additionally, the chloroplast experienced disorganization, including the de-stacking of thylakoids (**Figure 3K**) followed by the release of starch grains into the cytosol (**Figure 3L**). However, this release may occur before full chloroplast disintegration, with only the plastid envelope being partially disrupted (**Figures 3M–O**).

### 3.3 Carbohydrate metabolism of raspberries modulated by rust, post-formed defense mechanism in raspberries and structural alterations

**Figure 4:**
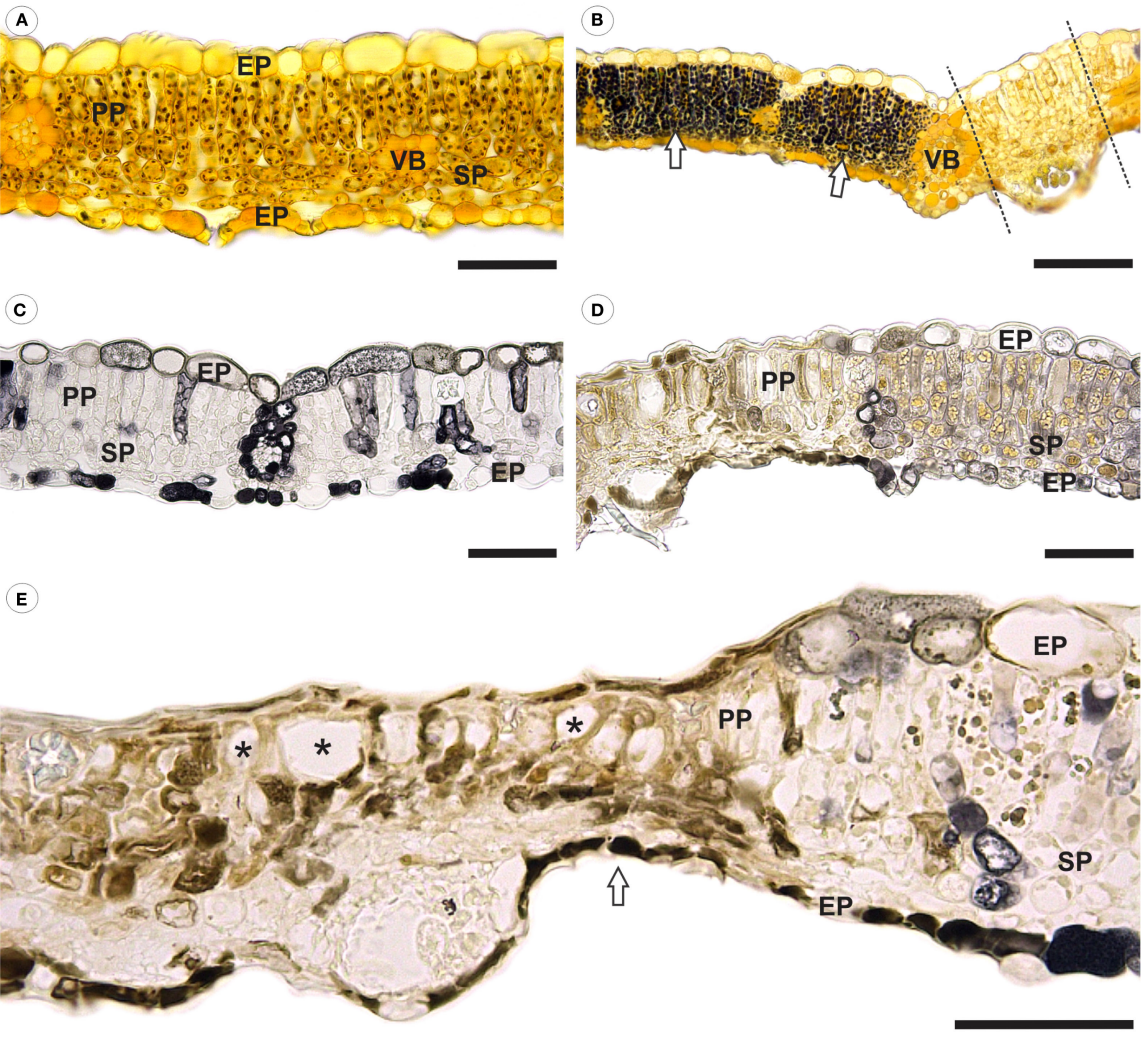
Bright field micrographs of cross-sections of healthy and *Aculeastrum americanum*-inoculated raspberry leaves subjected to histochemical analyses. **(A)** Healthy and **(B)** inoculated leaves after reaction with iodinated zinc chloride at 14 days after inoculation (DAI). In **(B)**, there is an intense reaction to starch (dark-brown/black spots) in the mesophyll cells in areas flanking a pustule (arrows). Dashed lines delimit the region of the pustule. **(C)** Healthy and **(D, E)** inoculated leaves fixed in ferrous sulfate solution in formalin at 21 DAI, showing a higher accumulation of phenolic compounds in inoculated leaves. **(E)** Necrosis and collapse of epidermal and mesophyll cells resulting in a marked reduction in the overall leaf thickness in the damaged area (arrow). Large spaces (*) in palisade parenchyma due to cell collapse. EP, epidermis; PP, palisade parenchyma; SP, spongy parenchyma; VB, vascular bundle. Scale bars: 50 μm **(A, C–E)**; 100 μm **(B)**.

Healthy leaves exhibited a typical starch reaction to iodinated zinc chloride solution, as indicated by dark-brown spots loosely scattered throughout the mesophyll cells cytoplasm (**Figure 4A**). In contrast, inoculated leaves showed a stronger starch reaction in mesophyll cells, particularly in areas flanking pustules (**Figure 4B**). Notably, no starch reaction was detected in the parenchyma cells located directly beneath the pustules (**Figure 4B**).

In leaves fixed with a ferrous sulfate solution in formalin, pre-formed phenolic compounds were detected in the epidermal, mesophyll, and vascular bundle cells (**Figure 4C**). In contrast, a higher accumulation of phenolic compounds was observed in areas where the pathogen was present (**Figures 4D, E**). Additionally, structural changes were noted in inoculated leaves, including the collapse of epidermal cells on both the adaxial and abaxial side. Similar alterations were observed in both spongy and palisade parenchyma cells, leading to the formation of large intercellular spaces in the palisade parenchyma (**Figures 4D, E**). These changes resulted in necrosis and a significant reduction in overall leaf thickness in the affected area (**Figure 4E**).

**Figure 5:**
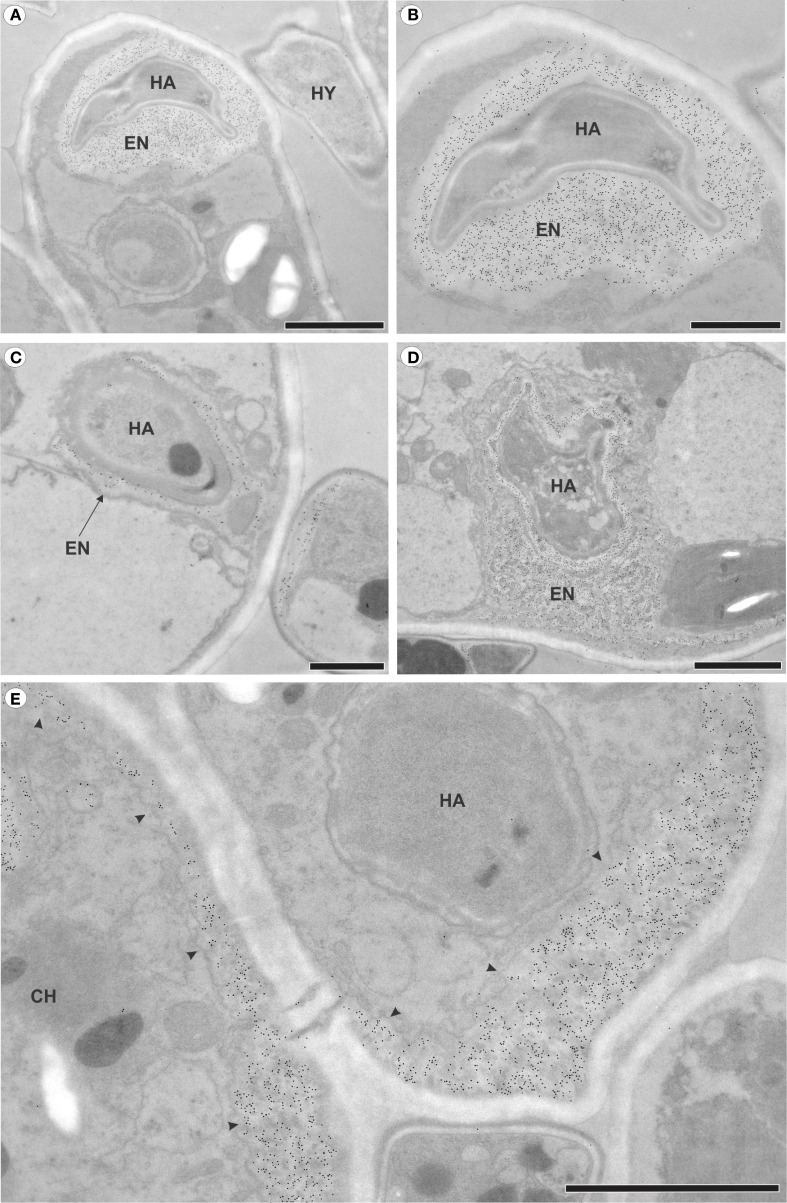
Transmission electron micrographs of mesophyll cells from raspberry leaves of inoculated plants with *Aculeastrum americanum* at 7 days after inoculation (DAI), showing callose detection via immunogold labeling. **(A)** Deposits of callose in the area where the fungus penetrates. **(B)** Detail of the haustorium of the fungus in **(A)**, surrounded by callose. **(C)** Partially encased haustorium. **(D)** Haustorium completely surrounded by callose. **(E)** Deposits of callose (arrowheads) in areas distant from the fungus penetration site. CH, chloroplast; EN, haustorial encasement; HA, haustoria; HY, hyphae. Scale bars: 1 μm **(B, C)**; 2 μm **(A, D, E)**.

The analysis of immunogold labeling for callose (**Figures 5A–E**) revealed deposits of 1,3-ß-glucan-containing material at the sites of fungal penetration (**Figures 5A, B**). Some haustoria were partially surrounded by this material (**Figure 5C**), while others were completely encased by a layer of encasing material (**Figure 5D**). Additionally, deposits were observed along the cell wall of infected cells (**Figure 5E**).

### 3.4 Reproduction

**Figure 6:**
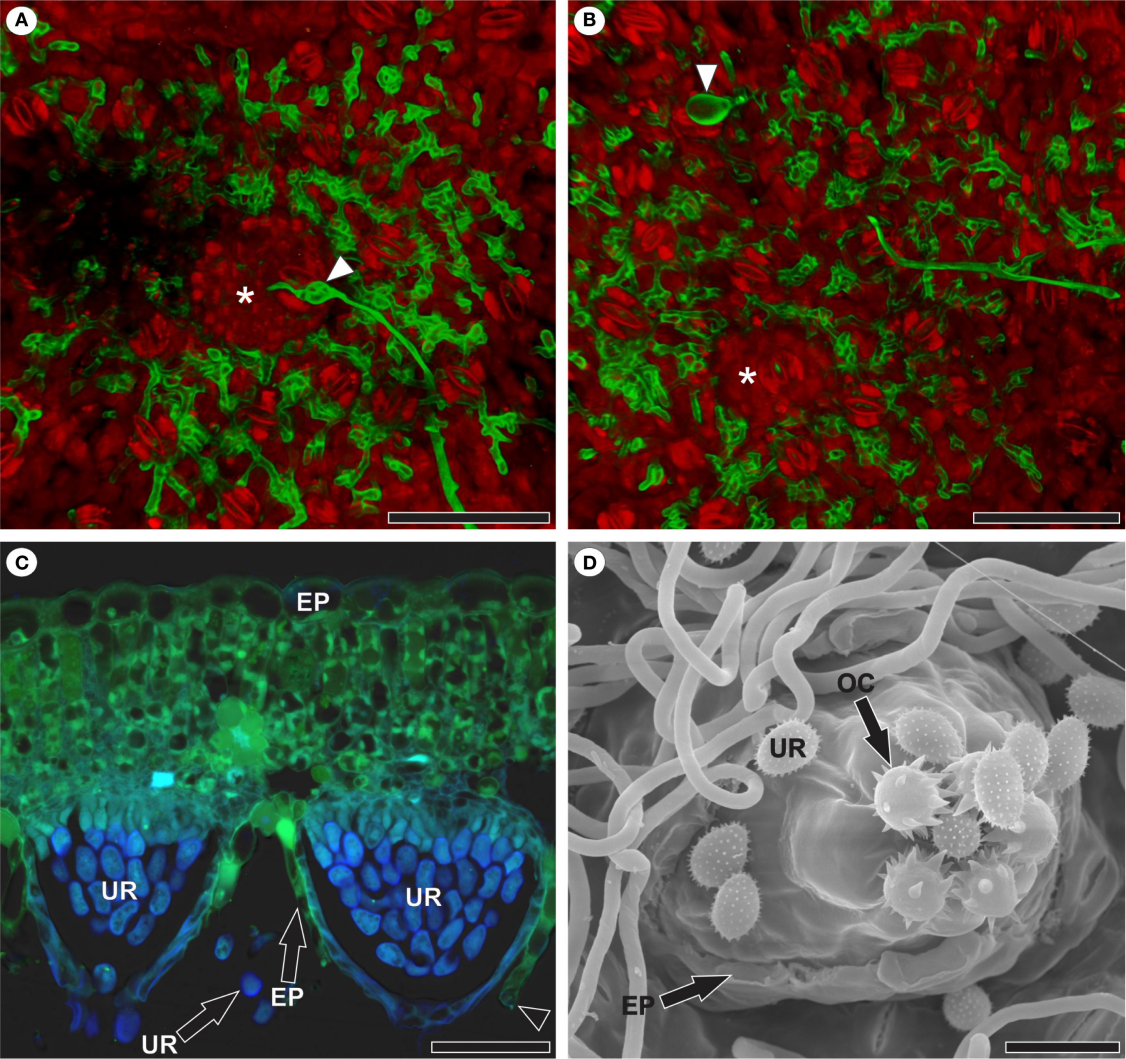
Reproduction of *Aculeastrum americanum* in raspberry leaves. **(A, B)** Confocal micrographs. **(C)** Fluorescence micrograph. **(D)** Scanning electron micrograph. **(A)** Uredinial primordium (*) in the substomatal chamber of the stoma through which penetration occurred as evidenced by appressorium (arrowhead) at 7 days after inoculation (DAI). **(B)** Uredinial primordium (*) in the substomatal chamber of a stoma nearby to the stoma in which appressorium (arrowhead) was observed and penetration occurred at 7 DAI. False colors in channel overlay image **(A, B)** represent WGA Alexa Fluor 488 signal (green) and propidium iodide signal (red). **(C)** Uredinia after rupture of the host leaf epidermis at 14 DAI. Note the presence of a guard cell in the disrupted epidermis (arrowhead) and autofluorescence of urediniospores with DAPI filter (light blue). Micrograph acquired using WGA Alexa Fluor 488 with a 5 L filter (460–500 nm excitation; 515–485 nm emission) and DAPI filter (340–360 nm excitation; LP 425 nm emission). **(D)** Abaxial leaf surface showing uredinia after rupture of the host leaf epidermis and the beginning of sporulation at 14 DAI. Note that the apex of the uredinia features ornamented ostiolar cells that define the opening through which the urediniospores are released. EP, epidermis; OC, ostiolar cell; UR, urediniospore. Scale bars: 50 μm **(A–D)**.

The formation of the uredinial primordium occurred in the substomatal chamber of the stoma through which penetration took place (**Figure 6A**) or in the substomatal chamber of a nearby stoma (**Figure 6B**). Uredinia broke through the abaxial epidermis of the host leaf, emerged among the trichomes, and began sporulating (**Figures 6C, D**). The apex of the uredinia featured ornamented ostiolar cells that define the opening through which the urediniospores are released (**Figure 6D**).

## 4 Discussion

Histopathological investigations of plant-pathogen interactions are crucial for addressing emerging plant diseases, particularly those affecting food crops. Here, we elucidate the infection, colonization, and reproduction processes of *Aculeastrum americanum* on raspberry leaves. Additionally, we provide new insights into chloroplast-pathogen interactions and, for the first time, we are able to demonstrate the formation of more than one haustorium within a single host cell by a rust.

Following the germination of *A. americanum* urediniospores, elongated and occasionally branched germ tubes are formed. The combination of a long and branched germ tube suggests enhanced efficiency in exploring the leaf surface and may indicate that this is a rust fungus that penetrates through stomata, as suggested by Hunt (1968). In *R. idaeus*, stomata are restricted to the abaxial leaf surface (Dias et al., 2023), where *A. americanum* exclusively forms appressoria and penetrates into the leaf, as reported for other rust pathosystems (Anikster et al., 2004; Boshoff et al., 2024; Leonard and Szabo, 2005; Noshad et al., 2023; Patton and Johnson, 1970). On the other hand, fungi that penetrate directly through the cuticle and epidermal cell wall generally form short, unbranched germ tubes, reflecting their low specificity in selecting penetration sites (Adendorff and Rijkenberg, 2000). Some species can employ both entry strategies, such as *Austropuccinia psidii* (Yong et al., 2019) and *Phakopsora euvitis* (Rasera et al., 2019); however, in the pathosystems described in these studies, stomatal entry is extremely rare and accounts for only a minor fraction of total appressorial penetrations.

Fungal hyphae of *A. americanum* developed within substomatal chambers and intercellular spaces of the mesophyll. They were absent from vascular tissues, unlike *Cronartium ribicola*, which invades vascular tissues in white pine (Jurgens et al., 2003), and *Puccinia horiana*, which colonizes xylem cells in the crown of chrysanthemum plants (Bonde et al., 2015). As colonization progresses*, A. americanum* grows intracellularly, forming a single haustorium or, as reported for rust for the first time in this study and supported by the presence of both a neck and a haustorial body in each haustorium, two haustoria per cell. The occurrence of two haustoria per host cell was observed in both epidermal and palisade parenchyma cells and may increase the pathogen’s interaction with the host, thereby enhancing its nutrient acquisition. Furthermore, we propose that this may enhance the pathogen’s ability to suppress host immunity and manipulate the host by creating two fronts of attack, thereby forcing the host cell to divide its defense resources. Although *Puccinia striiformis* f. sp. *tritici* has been reported to form two haustoria within cells of the wheat cultivar Gemmieza-11 (El-Sharkawy et al., 2024), the image may actually represent different sections of a single haustorium, as haustorial bodies can be sectioned at varying angles during sample preparation. The absence of two clearly defined necks in the observed cell further challenges the interpretation that these are distinct haustoria. In addition, the haustorium of *P. striiformis* is initially spherical and later becomes apically branched (Sørensen et al., 2012).

In infected raspberry cells, chloroplasts were positioned around fungus haustoria, similarly to what was observed in *Nicotiana benthamiana* cells infected by *Phytophthora infestans* (Savage et al., 2021). The chloroplast is a key component of early immune responses (De Torres Zabala et al., 2015), deactivates photosynthesis and produces microbial compounds, including hormones and secondary messengers, when activated by the plant immune system (Savage et al., 2021; Serrano et al., 2016). As part of their strategy for successful infection, pathogens can target chloroplasts and suppress their defensive functions (Xu et al., 2019). However, it remains unclear whether the association between chloroplasts and haustoria represents a plant defense mechanism or a pathogen virulence strategy (Savage et al., 2021). The authors proposed that the association of chloroplasts to haustoria could enhance the effectiveness of chloroplast-derived immune compounds and potentially trigger additional immune signaling. Nevertheless, they acknowledge that the association might also favor the pathogen, possibly by facilitating its nutrition. In a recent study, defense-related membrane contact sites were identified, specifically a membrane-anchoring complex between the outer chloroplast envelope protein CHUP1 and the extra-haustorial membrane-associated protein KAC1, which surrounds the haustorium of *P. infestans* in *N. benthamiana* (Yuen et al., 2025). According to the authors, this anchoring complex at pathogen penetration sites may contribute to the targeted delivery of defense components to the infection interface. In *R. idaeus*, the formation of more than one haustorium per host cell may compromise the immune response, as the number of chloroplasts associated with each haustorium tends to be lower compared to host cells containing only a single haustorium.

Chloroplasts also lose their autofluorescence in areas colonized by *A. americanum*, which likely reflects a reduction in chlorophyll content, a pigment known to be sensitive to biotic stress and typically decreasing during disease development (Cheaib and Killiny, 2025). Unlike the chloroplasts in *N. benthamiana* leaves infected by *P. infestans*, which remain intact (Savage et al., 2021), chloroplasts in raspberry leaves exhibited a series of alterations. This explains the drastic reduction in leaf gas exchange in plants infected by *A. americanum* (Ribeiro and Spósito, 2022). Initially, membrane vesiculation was observed, followed by thylakoid de-stacking, as reported in leaves *Triticum aestivum* infected by *Puccinia striiformis* (Aldesuquy et al., 2000), and ultimately by the structural disintegration of the chloroplasts, as observed in leaves of *Vitis labrusca* cv. Niagara Rosada infected with *Phakopsora euvitis* (Nogueira Júnior et al., 2017). Starch grains are released into the cytosol during chloroplast disintegration, a process that can begin even before the complete disruption of the plastid envelope. Once in the cytosol, they are consumed by the fungus, as no starch reaction to iodinated zinc chloride’s solution was observed in the mesophyll adjacent to the pustule. Fungi can hydrolyze starch through the action of amylases, enabling them to utilize this polymer as a carbon source (Goulet and Saville, 2017). Consistent with our results, Nogueira Júnior et al. (2017) reported the near absence of starch in mesophyll cells adjacent to the pustules in grapevine rust.

On the other hand, a substantial difference in starch was observed in regions flanking the pustules, with a stronger reaction to iodinated zinc chloride’s solution compared to healthy leaves, indicating that *A. americanum* can alter the metabolism of *Rubus idaeus* to its advantage. Several studies of plant-pathogen interaction have shown that the starch dynamic in leaves is altered (Chou et al., 2000; Gamm et al., 2011; Nogueira Júnior et al., 2017; Scholes and Farrar, 1987). Biotrophic pathogens can redirect host sugars to their needs by manipulating carbohydrate metabolism (Zadoks and Schein, 1979), causing infection sites to accumulate photosynthetic products at the expense of other plant regions (Long et al., 1975; Cheaib and Killiny, 2025). Indeed, in grapevines infected by *Phakopsora euvitis*, the accumulation of starch in leaves, like that observed in the present study, occurred to the detriment of starch accumulation in roots (Nogueira Júnior et al., 2017).

The accumulation of phenolic compounds has also been observed in areas where the pathogen was present, in contrast to healthy raspberry leaves that only present constitutive phenolic compounds (Dias et al., 2023). Phenolic compounds are well known for their antimicrobial activity (Lygin et al., 2009; Osbourn, 1996). A reduction in their biosynthesis or alteration in phenol pattern can compromise host defense and allow higher infection, as proposed by Rasera et al. (2024), who observed a higher number of pustules in grapevine rust under high temperature. The phenol accumulation in infected raspberry leaves corroborates findings from other rust pathosystems (El-Sharkawy et al., 2024; Jurgens et al., 2003; Kalisz et al., 2015; Lu et al., 2017; Lygin et al., 2009; Ullah et al., 2017).

In addition to biochemical changes, structural alterations were observed in the leaf, including the collapse of epidermal and parenchyma cells, leading to the formation of large intercellular spaces in the palisade parenchyma, a visible reduction in the overall leaf thickness in the damaged area and necrosis. Interestingly, rust-causing fungi are biotrophic pathogens which need living plant cells, characterized by causing minimal damage to host cells (Mendgen and Hahn, 2002). We cannot rule out the possibility that cellular collapse and subsequent necrosis may be a hypersensitive response (Coll et al., 2011), aiming to restrict fungal growth (Beardmore et al., 1983; Niks and Rubiales, 2002). Necrosis was also observed in grapevine leaves infected by *Neophysopella tropicalis* (Rasera et al., 2023). According to the authors, necrosis may have been favored by the rapid foliar colonization of the pathogen, followed by a delayed defensive response from the plant, possibly related to the short evolutionary period of coexistence between host and pathogen. All structural and ultrastructural changes observed in raspberry leaves infected by *A. americanum* may explain the early defoliation associated with this pathosystem (Nelson, 2011; Hofer et al., 2025).

Another defense mechanism for raspberry against *A. americanum* involves the deposition of 1,3-ß-glucan-containing material at fungal penetration sites, partially or completely encasing the haustoria. Callose acts as a physical and chemical barrier, playing a key role in the plant defense response to pathogen invasion (Wang et al., 2021). The deposition occurs between the plasma membrane and the cell wall (Voigt, 2014), and can partially or completely encase haustoria (Underwood, 2012), as observed in this study. In addition, it extended along the cell walls of infected cells, reinforcing them. Callose deposition partially encasing the haustoria has been described in leaves of *Vitis labrusca* cv. Niagara Rosada infected by *Phakopsora euvitis* (Rasera et al., 2019), as well as in different wheat cultivars infected by *Puccinia striiformis* (Kang et al., 2002).

Despite the defense mechanisms, such as callose production and accumulation of phenolic compounds, which can even delay pathogen colonization, raspberry leaves are unable to prevent successful colonization by *A. americanum*, which proceeds to reproduction. Initially, uredinial primordia develop within the substomatal chamber of the stoma through which penetration occurs or in the substomatal chamber of nearby stomata. These structures arise from the aggregation of hyphae and are characterized by compact masses of fungal cells (Baka, 2023). Subsequently, the primordium develops into an uredinium, in which urediniospores are formed (Hughes, 1970). These spores are released when the uredinium ruptures the host epidermis, typically occurring seven days after the onset of infection (Ribeiro and Spósito, 2022). After dispersal, primarily driven by wind and rain, as observed in studies of *Thekopsora areolata* (Zhang et al., 2022), the urediniospores can initiate new infections.

In conclusion, histopathological analyses of *A. americanum* on *R. idaeus* cv. Heritage, provided the first microscopic evidence, in rust fungi, of the formation of two haustoria, each with a neck and haustorial body, within a single host cell. Our findings also provide insights into the chloroplast-pathogen interaction, as chloroplasts were observed in close association with haustoria in infected cells and exhibited a series of alterations. Although infected leaves produce defense substances such as callose and phenolic compounds, raspberry leaves are unable to prevent successful colonization. Cell collapse and necrosis were also observed, which, together with the ultrastructural alterations, may help explain the early defoliation seen in raspberry plants infected by *A. americanum*. While considerable insight has been gained into the chloroplast-pathogen interaction in late leaf rust, further investigation into this process in other biotrophic pathogens, particularly those affecting food crops, such as rusts, is essential to enhance our understanding of this relationship.

## Data Availability

The original contributions presented in the study are included in the article/supplementary material. Further inquiries can be directed to the corresponding authors.
